# Barriers to Routine Use of Ultrasound-Guided Fascia Iliaca Blocks in High and Low-Resource Emergency Departments

**DOI:** 10.7759/cureus.117410

**Published:** 2026-10-03

**Authors:** Heather A Brown, Caroline Brady, Halley Alberts, Limbani Mapata, Singatiya Chikumbanje, Laura Nolting

**Affiliations:** 1 Emergency Medicine, Prisma Health Midlands, Columbia, USA; 2 Emergency Medicine, Queen Elizabeth Central Hospital, Blantyre, MWI; 3 Anesthesia and Critical Care, Queen Elizabeth Central Hospital, Blantyre, MWI

**Keywords:** emergency medicine, fascia iliaca block, hip fracture, regional anesthesia, ultrasound-guided procedure

## Abstract

Background

Ultrasound-guided regional anesthesia, such as fascia iliaca (FI) blocks, represents an alternative pain management strategy with decreased risk of oversedation and respiratory depression for emergency department patients.

Methodology

This is a two-site, descriptive, 12-month follow-up survey to evaluate FI block uptake and perceived barriers after training resident physicians at two academic training sites: an urban academic tertiary center in the United States and a resource-limited referral hospital in Malawi.

Results

In total, 34 participants from the US site and seven participants from the Malawi site completed the survey, with response rates of 87% and 28%, respectively. Nearly all participants agreed that FI blocks were useful and improved patient care (median = 4 on a five-point Likert scale), and the majority reported confidence in performing the procedure. Despite this, most participants had not performed an FI block in the preceding 12 months (62% US participants and 57% Malawian participants). Perceived barriers were rated with “time to perform the procedure” ranking the highest among US participants and “time to perform the procedure” and “patient disinterest/refusal” ranking the highest for Malawian participants. Lack of equipment or supervision was commonly noted as a reason Malawian participants had considered but not performed an FI block, but not for US participants. Patient comorbidities (25%) and orthopedic reluctance (20%) were reasons US participants had considered but not performed the procedure, but were not noted by Malawian participants.

Conclusions

Reported barriers varied according to location, and the findings suggest that barriers are multifactorial and highly context-dependent.

## Introduction

Pain is a common complaint in the emergency department (ED), and parenteral medications, particularly opioids, have historically been a mainstay of acute pain management. The use of opioid pain medications as first-line pain management has many disadvantages, including respiratory depression, oversedation, interactions with other medications, and the potential for addiction [1]. Undertreatment of pain is particularly common in low- and middle-income countries where advanced airway management may not be readily available if respiratory depression occurs as a side effect of opioids [2,3].

In recent years, attention has been given to the benefits of utilizing multimodal pain control in the ED, specifically ultrasound-guided regional anesthesia (UGRA) [4-6]. Professional organizations and emergency medicine (EM) educators increasingly recognize regional anesthesia as an important skill for emergency physicians, and regional anesthesia has been incorporated into EM educational curricula and proposed procedural competency expectations [7-10].

Patients with hip fractures, in particular, can greatly benefit from pain management with UGRA. Each year in the United States, over 300,000 elderly patients are hospitalized for hip fractures [11]. Elderly patients are particularly at risk for oversedation and respiratory depression. UGRA offers an alternative pain management strategy without the side effects of opioids. Additionally, UGRA decreases the risk of delirium in elderly patients with hip fracture compared to standard treatment [12]. The fascia iliaca (FI) block is especially safe as it is a compartment block that relies on diffusion of the anesthetic under the fascia surrounding the nerves within the compartment, and the needle does not approach the femoral vessels or nerve during the procedure [13]. However, there is more than one technique for performing FI blocks, as well as variation in the exact way ultrasound is utilized during the procedure, which can impact procedural time, safety, and duration of pain relief [13]. FI blocks have been shown to provide pain relief for up to eight hours with rare complications and decrease the need for parenteral pain medication [14,15].

While the safety and efficacy of UGRA performed by emergency physicians for managing pain is well established, nerve blocks are still underutilized in the ED setting [14-16]. Despite training and credentialing efforts, many studies have shown difficulty in translating knowledge to practice, with often low to modest skill uptake after training [17-19]. The purpose of this study was to describe the perceived utility, procedural uptake, and barriers to the routine use of FI blocks in the ED after physician training at two distinct implementation settings with different resources and practice patterns: a resource-rich academic ED in the United States and a resource-limited tertiary teaching hospital in Malawi. Preliminary results of this study were presented in poster format at the International Conference of Emergency Medicine in Montreal, Canada, on May 26th, 2025.

## Materials and methods

Study setting

This is a two-site study evaluating FI block uptake and perceived barriers after targeted and site-specific physician training in two markedly different clinical environments utilizing self-reported survey data. The US site is a large urban tertiary care center with an annual volume of 110,000 emergency patients in the Southeast United States with a long-standing EM residency. The hospital is a level one trauma center and has hosted an EM ultrasound fellowship since 2006. The Malawi site is the largest hospital in the country, located in a large urban center, and is the primary major teaching hospital and referral center for the country. EM is a new specialty in Malawi, and the medical school began training the country’s first EM residents in 2020. At the time of the study, there were four EM-trained faculty working at the site, and clinical ultrasound supervision was limited compared to the US site.

In total, 39 EM residents at the US training site received longitudinal, recurrent training in performing FI blocks. Beginning in August 2021, all first-year residents received FI block training during their dedicated ultrasound rotation. Simultaneously, an Epic™ order set specific for ED nerve blocks and a procedure note template was built into the electronic health record (EHR). Nerve blocks were taught with a comprehensive approach, targeting the logistics of ED peripheral nerve blocks, documentation, medication administration and safety, and hands-on practice. During the rotation, residents received 1:1 training with the ultrasound faculty reviewing and simulating several highly utilized nerve blocks in the ED. Additionally, we integrated block training for all residents several times annually during regularly scheduled required didactic sessions. This included didactic lectures reinforcing all aspects of the FI block discussed above and simulation days with hands-on experience practicing blocks on phantoms.

At the Malawi site, 25 residents from multiple specialties (EM, surgery, anesthesia, orthopedics, internal medicine, radiology, and family medicine) were trained in FI blocks during a single, two-day, in-person ultrasound-guided procedure training that took place at the training hospital in April 2023 and has been described in detail elsewhere [20]. Due to the small size of the EM residency program (four residents in total), a multidisciplinary approach was taken to train additional residents from various specialties who treat patients that might benefit from the FI block. One hour of the course was dedicated to a lecture focusing on the general approach to ultrasound-guided nerve blocks and another hour specifically on the FI block. Another 1.5 hours of the course was dedicated to hands-on practice with the FI block trainers in a 3:1 instructor-to-learner ratio. Each learner was required to complete an observed structured clinical examination demonstrating competency in performing an FI block on the trainer before completion of the course. Additionally, due to a paucity of supervisory physician oversight, an on-call ultrasound team was maintained on site for six weeks after completion of training to assist with the procedure. The on-call team consisted of course instructors and faculty from the US site, many of whom regularly spend time working and teaching at the Malawian site. An EHR was not in use at the time of the training. A required paper checklist was created to ensure safety guards and proper documentation.

Participants at both sites were taught the infrainguinal approach to the FI block. This approach was chosen as the landmarks are easier to identify, especially in the elderly. The suprainguinal approach, while typically leading to more proximal spread of the anesthetic, relies on identifying the internal oblique muscle, which is often atrophied in elderly patients and difficult to visualize, potentially making procedure time longer and less safe with learners. The procedure teaching approach was standardized between the two sites and utilized the same instructors with only minor adaptations for resource availability. Block kits, containing all necessary equipment to perform an FI block, were created for both sites with all necessary materials and kept in the respective EDs (Appendix 1). Kit contents varied slightly between the two sites as contents were locally sourced to ensure sustainability. A short site-specific instructional video was made for each site and was always available for just-in-time review.

Study design

This study is an exploratory, two-site, descriptive implementation assessment utilizing survey data to evaluate participants’ perceptions of FI block utility, uptake, and barriers to routine use in the ED after focused training. In June 2023, an anonymous electronic survey was distributed electronically to all 39 US participants (Appendix 2). Study data for this site were collected and managed using REDCap electronic capture tools. All 25 Malawian participants were asked to attend one of four in-person follow-up sessions in April 2024 (12 months after the training course) during which a similar paper-based survey was distributed to participants (Appendix 2). These sessions were also used to review procedure logs and provide an additional opportunity to practice FI blocks on the phantoms under supervision. Because the clinical environments and implementation support differed between sites, follow-up survey instruments were adapted to each site. Both surveys assessed procedural uptake, frequency of considering but not performing FI blocks, perceived utility, and perceived barriers to use. Both surveys used a combination of yes/no, write-in, and five-point Likert scale questions. Because FI blocks were not being performed in either ED before the course and practical training, there was no established baseline performance rate against which post-intervention implementation could be compared.

Inclusion and exclusion criteria

All course participants were invited to participate in the survey. Participants who did not complete the course or were no longer working at the institutions were excluded.

Statistical analysis

Survey data from each site were collated, managed, and analyzed using Microsoft Excel version 16.101.3 (Microsoft Corporation, Redmond, WA, USA). Descriptive analysis was performed using means and medians. Given the small sample size and the substantial methodological heterogeneity between sites, direct statistical comparison of the sites was not performed.

Ethics statement

This study was reviewed by the Prisma Health Institutional Review Board and given an exempt determination (1958848-1) and approved by the University of Malawi, College of Medicine Research and Ethics Committee (approval number: P.04/23/4056).

## Results

From the US site, 34 of 39 participants completed the survey, with a response rate of 87%. From the Malawi site, seven of 25 participants (four EM and three anesthesia residents) completed the survey, with a response rate of 28%. On a five-point Likert scale, nearly all participants at both sites agreed that FI blocks were useful and improved patient care (median = 4). The majority of participants reported confidence in performing the procedure: 68% (n = 23) of US participants and 100% (n = 7) of Malawian participants. Despite this overall high confidence and reported utility, uptake of the skill was low, with only 38% (n = 13) of US participants and 42% (n = 3) of Malawian participants having performed at least one FI block in the 12-month follow-up period. Perceived barriers to performing an FI block were rated on a five-point Likert scale, with “time to perform the procedure” ranking the highest among US participants (median = 4) and “time to perform the procedure” and “patient disinterest/refusal” ranking the highest for Malawian participants (median = 3) (Figure 1).

**Figure 1:**
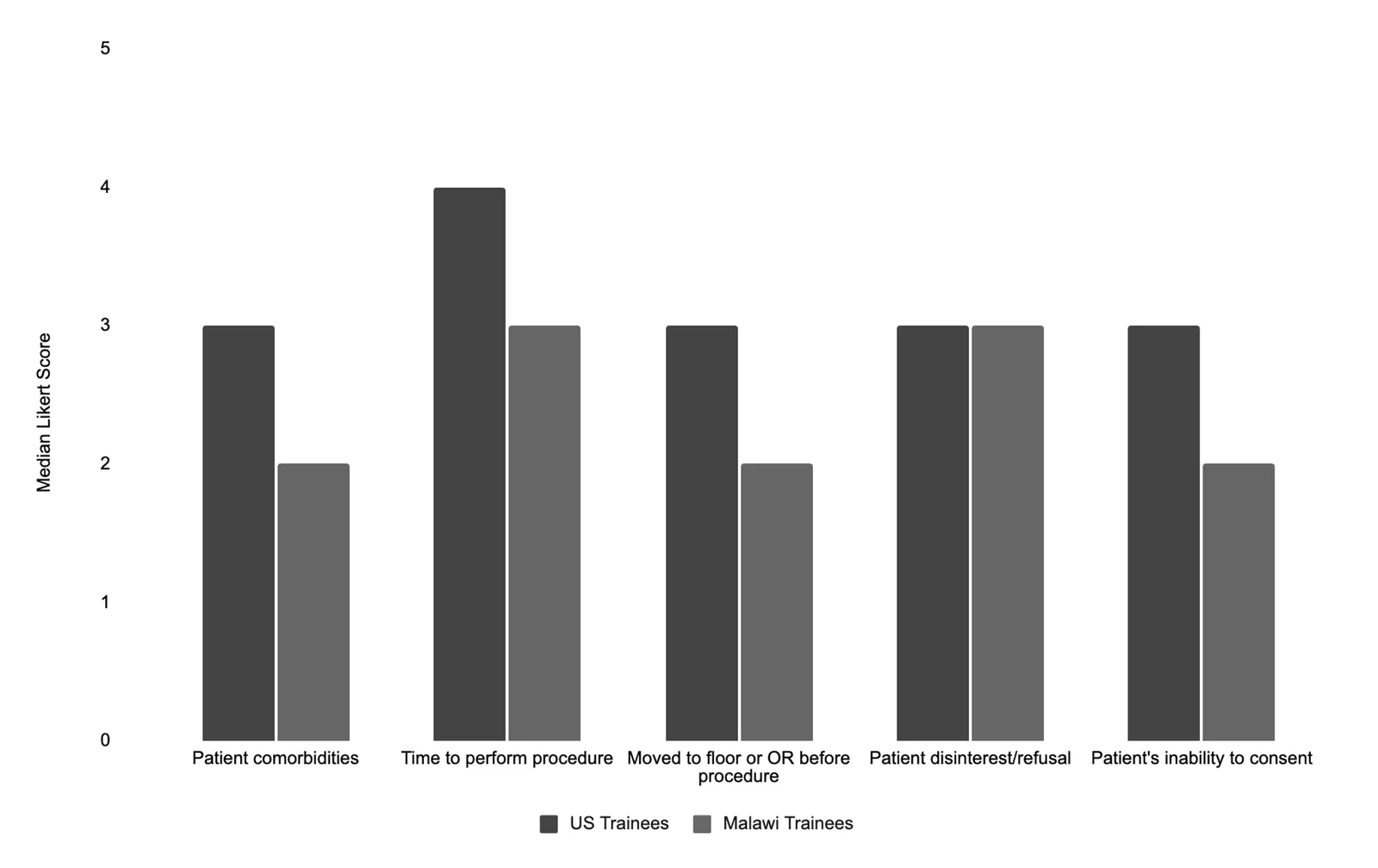
Perceived barriers to performing fascia iliaca blocks.

Most US participants “rarely” (median = 2) considered but did not perform an FI block, while most Malawian participants answered “sometimes” (median = 3) to the same question. Overall, 20 (59%) US participants reported “considering but not performing a block.” Reasons for considering but not performing a block were provided in a “check all that apply” answer format with an “other” write-in option. A total of 30 reasons were selected, including five “other” options, which all fell into the category of lack of time to perform the procedure (Table 1). Moreover, five (71%) Malawian participants reported considering but not performing a block. Malawian participants were asked for reasons for considering but not performing a block in a write-in response only. Write-in responses fell into the following three categories: lack of equipment or supervision, the alternative seemed easier or better, and lack of confidence (Table 1).

**Table 1:** Reasons for considering but not performing a fascia iliaca block by training site.

	US (n)	US (%)	Malawi (n)	Malawi (%)
Pain well controlled	8	26.6%	-	-
Lack of time	5	16.6%	-	-
Contraindication	5	13.3%	-	-
Orthopedic reluctance	4	13.3%	-	-
Emergency medicine attending reluctance	3	10%	-	-
Patient refusal	3	10%	-	-
Inability to consent	2	6.6%	-	-
Lack of equipment or supervision		-	2	40%
Alternative seemed easier/better		-	2	40%
Lack of confidence	--		1	20%

## Discussion

The use of regional anesthesia in the ED has expanded significantly over the last decade as emergency physicians increasingly seek effective, opioid-sparing approaches to pain management [7]. Despite a growing body of literature suggesting that regional anesthesia is a practical and valuable component of contemporary EM practice, UGRA remains under-utilized [14,16]. Few studies have directly evaluated perceived barriers to UGRA after formalized training and implementation, and, to our knowledge, there are no previous multi-site studies looking at two academic sites with such different resource capabilities [18,21-23]. In this two-site study, physicians at both sites perceived FI blocks as useful, yet routine utilization remained limited at both sites despite major differences in resource availability and training approaches. Because physicians’ perceptions of the clinical utility, feasibility, and applicability of FI blocks may directly influence their likelihood of incorporating the technique into practice, investigating these perceptions is an important component of identifying bias as a potential barrier. While the heterogeneity of the two sites and training approaches make direct comparison difficult, results from both sites provided valuable insight into the challenges bedside physicians face translating training into practice for UGRA.

The Malawi respondents perceived FI blocks as clinically valuable and expressed substantial interest in expanding their peripheral nerve block skillset. Qualitative responses identified equipment malfunction or limited equipment availability and insufficient access to procedural supervision as the most common reasons for not performing an FI block. Respondents reported high confidence in performing the skill; however, lack of confidence was still identified as a reason for not performing the block. This discrepancy may indicate that reported confidence reflected perceived theoretical understanding of the procedure rather than confidence in independently performing the block in a real-world clinical setting. These results imply that global confidence ratings may inadequately capture the multifaceted nature of procedural competency required in the ED. Future survey instruments should therefore differentiate between knowledge-based confidence, technical confidence in simulation or model-based practice, supervised clinical confidence, and confidence in independently performing the procedure in routine patient care.

Malawian respondents also cited lack of supervision as a barrier. The existing patient care infrastructure at this site involves attending physicians covering for long periods of time, with senior residents managing patients on-site and attending physicians supporting remotely. As such, the lack of direct supervision is not surprising, and future implementation should take this into consideration. The on-call ultrasound team, available in the six weeks following the course, was implemented to provide additional supervision for the procedure. Despite learners utilizing the ultrasound team for several other procedures, the team did not receive any calls for FI blocks in the given period. Collectively, these findings suggest that clinical confidence as well as system-level and organizational factors may significantly influence the adoption of regional anesthesia practices in resource-limited settings.

US respondents expressed similar positive attitudes regarding the utility of FI blocks. The majority of US respondents reported that the necessary equipment was readily accessible and expressed confidence in their ability to perform the FI block, suggesting that barriers to implementation extend beyond technical competency and equipment availability. Previous survey data in a high-resource setting has cited faculty experience as the most common barrier to performing UGRA in the ED, followed by interdepartmental politics/lack of collaboration [21]. Although stakeholders in the orthopedic departments at both sites were included from the earliest stages of FI block implementation, four US participants cited orthopedic reluctance as a reason they had considered performing a block on a specific patient but did not. Orthopedic reluctance was not cited as a barrier among Malawian participants. Although it is unclear why there was less perceived orthopedic reluctance in Malawi, it is possible that increased collaboration with orthopedics stemmed from the more integrated multidisciplinary training offered at this site. Comorbidities were a stronger deterrent for using UGRA in the United States when compared to Malawi. While specific comorbidities were not recorded, we hypothesize this was most likely related to the high number of patients on blood-thinning agents as well as higher rates of obesity in the United States, making identifying landmarks more difficult in these patients [24,25].

These findings further emphasize that successful implementation of regional anesthesia in the ED may depend not only on individual provider confidence and equipment access but also on interdisciplinary support, attending-level familiarity, and workflow feasibility within the ED setting. These collective findings are consistent with prior studies looking at barriers to FI block utilization [17,18]. In the United States, these results would suggest that improving attending physician procedural exposure and comfort, as well as finding ways to combat workflow time and patient load constraints, would increase regional anesthesia utilization in the ED. At resource-limited sites, next steps may include ensuring reliable ultrasound and supply availability, increasing clinical confidence in performing the procedure independently, and focusing on training local supervisors to assist when available. Additionally, learners at both sites were taught a single, standardized approach to perform the FI block, which the course instructors considered the safest and simplest approach. It is possible that procedural variations could decrease the time and overall procedural burden to the performing physician. Future implementation research should consider whether technically simplified ultrasound-guided approaches or other procedural optimizations could increase uptake while maintaining patient safety.

Limitations

There are several limitations to the study, including significant differences between sites in length of training, target training population, ongoing supervision of the procedure, implementation support, and variation in survey tools, making direct comparison of results between the two sites difficult. Another major limitation of the study is the low response rate for the Malawi site, likely due to the in-person nature of the follow-up when the survey was administered. Though the multidisciplinary approach may have had other benefits, it also increased the logistical challenges for administration of the in-person survey, and there was no remote option for trainees who might be located in another clinical setting at the time of evaluation. Course instructors felt it was important to have participants attend the follow-up sessions given the short nature of the course and limited formal follow-up. However, sending an electronic copy of the survey to participants who did not attend the in-person follow-up sessions could have increased the response rate.

Differences in implementation represent a limitation as well. The small numbers of trainees at the Malawi site and the multidisciplinary approach to patient care at this site were driving factors in the decision to expand to other departments in contrast to the US site, which only included EM trainees. It is unclear how participant specialty may have impacted the results and is further complicated by the follow-up survey in Malawi only being completed by emergency and anesthesia participants. The differences in the FI block training between the two sites also create a limitation. Due to the availability of instructors and the logistical requirements necessary for implementation, the Malawi site was a two-day course with procedural support through an on-call ultrasound team for six weeks following. The US site was a longitudinal approach utilizing their focused ultrasound elective for initial training with additional regularly scheduled didactic sessions throughout the year. These differences could have influenced utilization, but the current assessment tools were not designed to evaluate the difference in efficacy of the training courses.

Assessment tools differed between the two sites as well. Although both instruments assessed shared domains of FI block uptake, perceived utility, confidence, and barriers, several questions differed in wording, response format, and site-specific content. Additionally, the instruments included different site-specific questions reflecting local implementation support, such as EHR documentation in the United States and the on-call ultrasound team support in Malawi. Both survey instruments relied on self-reported data for both the number of procedures performed and confidence measurements, potentially introducing recall and reporting bias. More objective data, such as procedural log review or a validated measure of procedural confidence, could have given more accurate results. The surveys utilized do not provide a quantitative value assessing the number of opportunities for implementation. To provide a distinction between lack of opportunities and lack of implementation of the intervention, this value should be included in future studies.

Neither site had attending-specific training and had very different levels of attending physician availability for ongoing procedural supervision for participants after the training. As attending familiarity and comfort can greatly influence trainee utilization, this represents a limitation at both sites [21]. In Malawi, the training was made available to both residents and attendings, with a total of two attendings participating. In the United States, training materials, including videos and practice opportunities, were made available, but there is no record of the frequency of utilization of these materials.

## Conclusions

Resident physicians in both the large, urban academic tertiary center and the resource-limited referral hospital perceived FI blocks as clinically useful and beneficial to the patient. Despite this, the use of UGRA in the ED following focused training remained inconsistent, and the reported barriers to increased nerve block utilization varied according to location. These findings suggest that barriers to FI block implementation are multifactorial and highly context-dependent. Accordingly, FI block training programs should extend beyond technical instruction alone and incorporate strategies focused on improved workflow integration, longitudinal supervision and mentorship, institutional support, and development of sustainable procedural systems tailored to the local clinical environment.

## Appendices

Appendix 1: Fascia iliaca block kit contents

**Table 2:** Fascia iliaca block kit contents by location. *: Ropivacaine was stored separately from the block kit.

United States block kit content	Malawi block kit content
Chloraprep	Betadine
Sterile ultrasound probe cover	Sterile ultrasound probe cover
Skin marker	Skin marker
Block needle with attached extension tube	Quincke spinal needle
50 cc syringe	Extension tube
Ropivacaine 0.2% (40 mL)*	50 cc syringe
	Bupivacaine 0.5% (20 mL)

Appendix 2: Survey questions

1.1 US Participant Electronic Survey Questions

1. In the last 12 months, how many fascia iliaca (FI) blocks have you performed?

(a)    0

(b)    1-3

(c)    4-6

(d)    6+

2. In the last 12 months, how often have you considered performing a fascia iliaca (FI) block but did not?

(1)   Never

(2)    Rarely

(3)    Sometimes

(4)    Often

(5)    Very often

3. Did you use the nerve block dot phrase in Epic to record every FI block?

(a)    Yes

(b)    No

(c)    Sometimes

(d)    n/a

4. Did you use the POCUS order in Epic for every FI block you performed?

(a)    Yes

(b)    No

(c)    Sometimes

(d)    n/a

5. Do you feel like supplies/equipment for the FI block are easily accessible?

(a)    Yes

(b)    No

6. Did you know the FI block has a premade kit?

(a)    Yes

(b)    No

7. Do you feel confident performing the steps for the FI block?

(a)    Yes

(b)    No

8. In the last 12 months, how often have you considered performing an FI block on a patient but did not?

(1)    Never

(2)    Rarely

(3)    Sometimes

(4)    Often

(5)    Very often

9. If you considered doing an FI block and did not, what was the reason (check all that apply)?

Orthopedist told you not to

EM attending told you not to

Patient could not consent

Patient met a contraindication covered in the training

Patient’s pain was well controlled

Patient refused or did not want the block

Other:

10. In your personal experience, do you think the block is helpful for analgesia?

(a)    Yes

(b)    No

(c)    N/a

11. Patient comorbidities are a barrier to performing FI blocks

(1)   Strongly disagree

(2)    Disagree

(3)    Neutral

(4)    Agree

(5)    Strongly agree

12. The time it takes to perform the procedure is a barrier to performing FI blocks

(1)    Strongly disagree

(2)    Disagree

(3)    Neutral

(4)    Agree

(5)    Strongly agree

13. Time to OR or floor before block can be performed is a barrier

(1)   Strongly disagree

(2)   Disagree

(3)    Neutral

(4)    Agree

(5)    Strongly agree

14. Patient disinterest or refusal is a barrier to the FI block

(1) Strongly disagree

(2)    Disagree

(3)    Neutral

(4)    Agree

(5)    Strongly agree

15. Patient’s inability to consent is a barrier to performing FI blocks 

(1) Strongly disagree

(2)    Disagree

(3)    Neutral

(4)    Agree

(5)    Strongly agree

16. The FI block is a useful procedure that improves patient care

(1)   Strongly disagree

(2)   Disagree

(3)    Neutral

(4)    Agree

(5)    Strongly agree

17. I will continue to perform FI blocks once I graduate from residency

(1)    Strongly disagree

(2)    Disagree

(3)    Neutral

(4)    Agree

(5)    Strongly agree

1.2 Malawian Participants Paper Survey Questions

1. What is your specialty?  (Please circle one.)

Anesthesia

Emergency Medicine

Family Medicine

Internal Medicine

Orthopedics

Surgery

2. In the last 3 months, how many fascia iliaca (FI) blocks have you performed?    ______________

3. Did you record every block in your procedure log?

Yes

No

4. In the last 3 months, how often have you considered performing an FI block on a patient but did not?

(1)   Never

(2)    Rarely

(3)    Sometimes

(4)    Often

(5)    Very often

5. When you have considered performing an FI block and did not, what was/were the reason(s)?

6. Did you perform an FI block with the on-call procedural ultrasound call team during the 6 weeks post-course?

Yes

No

7. Did you perform an FI block with a consultant after the on-call procedural ultrasound call team was no longer available?

Yes

No

8. Have you independently performed an FI block?

Yes

No

9. Drug and equipment availability is a major barrier to performing FI blocks at our hospital.

1.     Strongly disagree

2.     Disagree

3.     Neutral

4.     Agree

5.     Strongly agree

10. Patient comorbidities are a major barrier to performing FI blocks at our hospital.

1.     Strongly disagree

2.     Disagree

3.     Neutral

4.     Agree

5.     Strongly agree

11. The time it takes to perform the procedure is a major barrier to performing FI blocks at our hospital.

1.     Strongly disagree

2.     Disagree

3.     Neutral

4.     Agree

5.     Strongly agree

12. Patient movement to the ward or operating theatre before a block can be performed is a major barrier to performing FI blocks at our hospital.

1.     Strongly disagree

2.     Disagree

3.     Neutral

4.     Agree

5.     Strongly agree

13. Patient refusal or disinterest in a nerve block is a major barrier to performing FI blocks at our hospital.

1.     Strongly disagree

2.     Disagree

3.     Neutral

4.     Agree

5.     Strongly agree

14. Patient’s inability to consent is a major barrier to performing FI blocks at our hospital.

1.     Strongly disagree

2.     Disagree

3.     Neutral

4.     Agree

5.     Strongly agree

15. The FI block is a useful procedure that improves patient care at our hospital.

1.     Strongly disagree

2.     Disagree

3.     Neutral

4.     Agree

5.     Strongly agree

16. Peripheral nerve blocks are an important skill for physicians to learn.

1.     Strongly disagree

2.     Disagree

3.     Neutral

4.     Agree

5.     Strongly agree

17. I would like to learn additional peripheral nerve blocks in the future.

1.     Strongly disagree

2.     Disagree

3.     Neutral

4.     Agree

5.     Strongly agree
